# Improving the effectiveness of point of care tests for malaria and anaemia: a qualitative study across three Ghanaian antenatal clinics

**DOI:** 10.1186/s12913-020-05274-7

**Published:** 2020-05-19

**Authors:** Tanith Palmer, Abiola O. Aiyenigba, Imelda Bates, Doris Dokua Okyere, Harry Tagbor, Gifty Dufie Ampofo

**Affiliations:** 1 Liverpool School of Tropical Medicine, Pembrooke Place, Liverpool, L3 5AQ UK; 2 School of Medicine, University of Health and Allied Sciences, PMB 31 Ho, Volta Region Ghana

**Keywords:** Antenatal care, Malaria and anaemia in pregnancy, Active participation, Rapid diagnostic test, Haemoglobin colour scale, LMIC, Ghana

## Abstract

**Background:**

Anaemia and malaria are both major contributors to maternal and child mortality, and morbidity, with some of the worst outcomes occurring in sub-Saharan Africa. Point of care tests (POCT), if used appropriately, provide a simple, inexpensive form of diagnostic testing, as a reliable alternative when laboratory tests are not readily available. In such resource limited settings, clinical staff tend to rely on symptom-based diagnosis and presumptive treatment. This study uses qualitative methods to identify the current practice of POCT use for malaria and anaemia, to explore the enablers and barriers to effective implementation of these POCT, and to determine how relationships between each of the stakeholder groups may impact on POCT use.

**Methods:**

Staff (clinical and laboratory) and patients (pregnant women) at three antenatal care facilities within the Ashanti Region of Ghana participated in interviews and focus group discussions (FGDs). An initial coding framework was developed based on the pre-defined objectives of the study. Thematic analysis was used to identify subthemes and categories within each of the key themes.

**Results:**

At the time data were collected all three facilities used malaria POCT either as an adjunct to microscopy, or as their only form of malaria testing. Although all three facilities were familiar with haemoglobin colour scale (HCS), none of the facilities used them routinely. Clinical staff perceived symptom-based diagnosis was a quick way to diagnosis because access to POCT during consultations was unreliable, but recognized disadvantages associated with symptom-based diagnosis.

Perceived advantages of malaria and anaemia POCT were user-friendliness, improved diagnosis and opportunity for patient engagement, as well as lower cost implication for patients. Perceived disadvantages included likelihood of missed diagnosis of mild anaemia, as well as likelihood of human error leading to in accurate diagnosis which could impact on patient trust. Poor communication and lack of trust between staff groups was also identified as a barrier to effective uptake of POCT.

**Conclusions:**

Consistent supply of POCT as well as staff training and staff and patient engagement, are fundamental to successful uptake of POCT for effective malaria and anaemia management.

## Background

Anaemia and malaria both contribute significantly to maternal and child mortality, and morbidity, with some of the worst outcomes occurring in sub-Saharan Africa. Worldwide, there are approximately 3.4 billion people at risk of being infected with malaria each year, but 92% of malaria deaths occur in Africa [1]. The prevalence of anaemia in pregnancy is approximately 40% in Africa [1, 2]. In sub-Saharan Africa, POCT have been used routinely to test for syphilis, hepatitis, HIV, presence of protein in urine during antenatal care. However, the methods of testing vary depending on standard practice within the local context. This article focuses on the use of POCT for malaria and anaemia diagnosis in resource limited antenatal care settings.

In resource limited settings where laboratory infrastructure may be lacking, clinical staff rely on symptom-based diagnosis and presumptive treatment. Presumptive treatment is the treatment of clinically suspected cases without confirmatory laboratory tests. Point of care tests (POCT), if used appropriately, provide a simple, inexpensive form of diagnostic testing, as a reliable alternative when laboratory tests are not readily available [3]. They allow users to generate results quickly, to provide prompt treatment with the aim of improved outcomes. POCT are available for both malaria and anaemia, for example, in the form of malaria rapid diagnostic tests (mRDTs) and the haemoglobin colour scale (HCS) respectively.

There is evidence to suggest that presumptive diagnosis has lower sensitivity and specificity when compared to POCT. For example, although the accuracy of mRDTs in field settings is reported to be lower than the minimum WHO guideline of 95% sensitivity and 90% specificity for all malaria species in laboratory conditions [4]; this demonstrates a higher efficiency than presumptive diagnosis. There is also evidence to suggest that 32–93% of patients in malaria endemic areas are falsely diagnosed with malaria [5]. Also, the estimated sensitivity and specificity of HCS are both 80%, compared with presumptive diagnosis (52% sensitivity, and 75% specificity) [6]. Symptoms that are used for symptom-based diagnosis are often unreliable and can result in unnecessary or wrong treatment of patients such as unnecessary blood transfusions and anti-malaria treatment leading to increased risk of blood borne infection and anti-malarial resistance, respectively [7].

Given the decline in malaria prevalence globally [8], misdiagnosis of people with fevers is likely to increase. The POCT have advantages, in comparison to symptom-based diagnosis, that can potentially improve diagnosis of malaria and anaemia, if standardised and used effectively. Regardless of increased global funding for POCT development, and their known benefits, healthcare staff in resource-limited settings may still rely on symptom-based diagnosis and presumptive treatment as their primary approach of management. This study utilises qualitative interviews with antenatal care staff, laboratory staff and antenatal attendees (pregnant women), in three antenatal clinics in Ghana, to identify the current practice of POCT use, to explore the enablers and barriers to effective implementation of POCT, and to determine how relationships between each of the stakeholder groups may impact on POCT use. Identifying the problems and exploring options to overcoming these challenges could provide a means of improving effective use of POCT in antenatal clinics (ANC).

## Methods

### Study site

Three antenatal care facilities within the Ejisu-Juaben Municipality and Sekyere-East District of the Ashanti Region of Ghana were selected to participate in the study from April to June 2015. The Sekyere-East district is predominantly rural while the Ejisu-Juaben municipality has semi-urban and rural areas. Antenatal facilities within the two districts had been identified and mapped as part of an earlier study which aimed to determine the effectiveness of pregnant women’s active participation in their antenatal care for the control of malaria and anaemia in pregnancy [9]. Researchers from the initial study provided recommendations on the selection of facilities to provide a diversity of contexts including type of facility, size and source of funding.

Two of the three facilities selected were government funded - one hospital and one health centre. The third facility was a privately funded maternity home. Antenatal care services on specific days as well as drop-in advice sessions were offered in all selected facilities. The average number of first-time attendees per month at the government hospital, government health centre and private maternity home were 153, 16 and 67 respectively [9]. All three facilities had laboratories and trained laboratory staff.

Rapid diagnostic tests were used routinely at all three facilities when testing for malaria. The HCS had been previously piloted in two of the three facilities to estimate haemoglobin values for anaemia screening as part of the initial study. A control facility from the earlier study was selected as the third facility for the current study to help explore whether there may be any differences in the perspectives regarding POCT between those which had used HCS and those which had not.

### Recruitment and participant selection

Interviews and focus group discussions (FGDs) were conducted amongst clinical and laboratory staff as well as pregnant women of each of the three facilities. The facility visits, which lasted approximately half a day, took place on days that antenatal clinics were known to run, and laboratory staff were also present. Each facility was visited twice at times that ensured the same laboratory and midwifery staff were present.

#### Laboratory staff and antenatal clinic staff

The three antenatal clinics were midwife led, with a combination of midwives and nurses offering services to pregnant women. Auxiliary staff included health assistants, health extension workers and ward assistants with varying levels of formal nursing training of 1 year or less to none but the development of knowledge and skills on the job during their employment. Clinical staff who were directly involved with requesting and/or performing malaria and anaemia testing were invited for interview (participants were purposively selected to maximise diversity in expertise and seniority). They consisted of midwives and midwife/ward assistants. Laboratory staff who were present at the facilities visited were also invited for interview.

#### Antenatal clinic attendees – pregnant women

All women aged 18 years and above, who were attending antenatal clinic appointments on the day of the facility visit, were invited to participate.

### Data collection

Data collection was undertaken by three qualitative researchers who had been involved in the initial study [9] using semi-structured interviews and FGDs; these are available as Additional files 1, 2, 3 and 4 (supplementary materials). Guides were developed to cover the scope of the research objectives and were informed by the findings of the initial study [9]. The data collection was done in two visits to each facility using interviews and FGDs which lasted 30 to 60 min, and 45 to 75 min, respectively. In total, nine FGDs and eleven interviews were conducted across all three facilities. FGDs amongst pregnant women consisted of two stages at each of the three health facilities visited, as well as one FGD amongst ANC and laboratory staff at each facility. Interviews amongst laboratory and ANC staff were stopped when the point of saturation was reached.

For clinical and laboratory staff, the first visit involved semi-structured interviews and explored topics in current practices for diagnosis and treatment of malaria and anaemia and their perceived advantages and disadvantages, communication and relationships between laboratory and antenatal staff, and usage and perceptions of POCT for anaemia and malaria. At the second visit, FGDs were conducted which explored barriers preventing the effective use of POCT, potential strategies for promoting change to ensure POCT effective use, and identification of key stakeholders necessary for implementing these changes. The purpose of the two visits was to explore differences between the clinical and laboratory staff groups, as well as their working relationships.

For the pregnant women, FGDs were conducted in each facility at both visits. This involved groups of 8 to 16 pregnant women who were only allowed to participate once to avoid their views being presented twice. Their perceptions and experiences of malaria and anaemia testing were explored as women from a variety of backgrounds but having in common pregnancy and sharing the same antenatal care services. In order to explore relationships between the pregnant women and clinical and laboratory staff, vignettes were used to elucidate how women would respond if treatment was prescribed by staff when they were aware that they had tested negative for anaemia or malaria. If a participant was not aware of POCT, a brief technical explanation was given to aid discussion.

### Data recording, analysis and ensuring quality and anonymity

Interviews were conducted in English and translated into the local language (Twi) by another member of the research team, as required. All FGDs were conducted in Twi to reduce the impact of language and cultural barriers. FGDs and interviews were audio recorded with permission from the participant(s) and were transcribed into English for analysis. Audio recordings were transcribed verbatim by the research team. Participants were assured that information would be gathered, processed and analysed in confidence; any quotes used would not be personally identifiable. All transcribed data were imported onto NVIVO 12 (windows version 12.5, 2019) to be analysed and interpreted through content analysis to develop research headings, categories and subcategories. Data was independently classified and analysed by two investigators (TP and AA) to enhance the credibility of the categorisation. To deepen insights and ensure findings reflected the research context, analysis of data was regularly reviewed by the research team who had carried out data collection.

A coding framework was initially developed by one researcher based on the pre-defined objectives of the study and elaborated by a preliminary review of the data to understand the narratives portrayed by participants. Themes were discussed and agreed with a second researcher. Inductive coding was used to identify subthemes within each of the key themes. Themes were illustrated with quotes which were also mapped into the framework. The quotes were labelled using generic job roles to ensure anonymity. FGD participants were assigned a letter to which personal information (e.g. age, education level) and quote transcription was attributed.

### Ethics

The study, including interview and FGD guides, participant information and consent forms, were approved by the ethics committees of the Liverpool School of Tropical Medicine UK, and the School of Medical Sciences in Kumasi Ghana, prior to the start of the study. Information regarding the study was explained verbally to participants prior to interview and FGDs. Participants were also provided with information sheets before seeking their consent using pre-prepared consent forms. Participants were informed that the information they provided would be collated, analysed and circulated and consent reaffirmed. All of this was explained in the local language whenever required.

## Results

Table 1 shows distribution of study participants. Eleven staff members across all sites agreed to be interviewed: seven midwifery staff and four laboratory staff. There were no refusals to participate. Quotes from staff are cited with clinical/laboratory and facility and no further details to maintain confidentiality; quotes from pregnant women are cited with age and facility used (Table 2).

**Table 1:** Demographic distribution of study participants (ANC and laboratory staff; pregnant women)

**Demographic distribution of staff [*N*** **= 11]**			
***Facility***	***Job Role***	***Length of Service at Facility***	
Government Hospital (GH)	Midwifery Officer	7 years	
	Senior Midwifery Officer	8 years (with previous experience elsewhere)	
	Deputy Head of Nursing	12 years (with considerable previous experience elsewhere)	
	Deputy Head of Laboratory Services	1.5 years (with 1.5 years’ experience elsewhere)	
	Laboratory Assistant	19 years (with previous experience elsewhere)	
Government Health Centre (GHC)	Midwifery officer	2 weeks (with previous experience elsewhere)	
	Principal Laboratory Assistant	13 years	
Private Maternity Clinic (PMC)	Ward Assistant	11 years	
	Midwife in-charge	21 years	
	Ward Assistant	2 years (with 1-year experience elsewhere)	
	Laboratory Scientist	13 years	
**Demographic distribution of pregnant women [*N*** **= 40]**			
***Facility***	***Characteristics***		***Count***
Government Hospital (GH) [*n* = 18]	Age group (years)	18–25	11
		26–30	3
		31–35	3
		> 35	1
	Gestational age (months)	0–3	1
		4–6	11
		> 6	6
	Level of education	None	0
		Primary	1
		Secondary	15
		Tertiary	2
Government Health Centre (GHC) [*n* = 14]	Age group (years)	18–25	8
		26–30	5
		31–35	0
		> 35	1
	Gestational age (months)	0–3	3
		4–6	2
		> 6	9
	Level of education	None	2
		Primary	3
		Secondary	9
		Tertiary	0
Private Maternity Clinic (PMC) [*n* = 8]	Age group (years)	18–25	3
		26–30	3
		31–35	0
		> 35	0
	Gestational age (months)	0–3	2
		4–6	3
		> 6	3
	Level of education	None	0
		Primary	2
		Secondary	6
		Tertiary	0

**Table 2:** Table showing listed verbatim quotes from participants (I = Interviewer; *R* = Respondent)

No	Setting	Participant description	Verbatim quote
1	FGD, GH	Laboratory and clinical staff	*“We only test for the falciparum. So, if we say…if the results come from the lab says it’s negative, we don’t test for the others so we wouldn’t know if it’s really negative for the others or it’s a different condition presenting like that we wouldn’t know...”*
2	FGD, GH	Laboratory and clinical staff	*“R: I think some work has been done but estimated about 7%, is that right?* *I: Between 6 and 7%, between 6 and 7%... How accurate do you think the RDTs are?* *R: I think that brand is also important. Some of them are very accurate in picking but some…err I don’t know. But some don’t usually give you the positive that you want but if you do the microscopy you realize the organisms are there, but you don’t get the report from the RDTs.”*
3	Interview, GH	Laboratory staff	*“That’s what they used to say that oh we checked conjunctiva; it is pale. We checked the palms; the patient is pale and your Hb is… your Hb is not corresponding to the patient. But you see, if it happens like that we can’t do otherwise. If you repeat the test and the machine gives you the same value, there’s nothing you can do. You have to write that way so it’s left to the clinician to judge.”*
4	Interview, GHC	Laboratory Manager	*“I: Do you think that the doctors here trust the RDTs?* *R: I don’t know anything about it whether they trust it or not.”*
5	Interview, GHC	Clinic staff	*“I- So someone could come to you with symptoms of anaemia but doesn’t actually have anaemia. Does that ever happen…?* *R – No* *I – …So those symptoms are always anaemia?* *R – Yes”*
6	Interview, GH	Laboratory staff	*“I think so because some people can be colour blind. If they use this their judgement will be different from what’s really happening… sometimes too the RDTs when you use them, when you use the RDTs there can be very faint lines. I might see it but you might not see it.”*
7	Interview, PMC	Clinical staff	*“The people from the insurance office came to talk with us about that. If a person’s test comes out not positive, we should not treat it but if we should treat, we are to write the reason why we are treating it.”*
8	Interview, GH	Laboratory staff	*“She doesn’t understand, that’s how I see it. Sometimes, it’s a different sickness. It can be a different sickness but not malaria.”*
9	Interview, PMC	Laboratory staff	*“Yeah that one is a problem because these, if you have fever or your temperature is high, not only malaria can cause that so there is a problem. That what we know. We have a problem…. I think even the teaching hospitals they are doing it so there’s a problem.”*
10	Interview, GH	Laboratory staff	*“Eheee, they bring the request, we do it, there’s no malaria, they still continue to treat; not for all patients anyway. But for some of them they still continue to treat. We always complain, we always talk about that. They are trying to stop but they still do it anyway.”*
11	Interview GH	Laboratory staff	*“… sometimes we ask the prescribers and the nurses to give us feedback…maybe within a week, within a day or two if they are getting some, a particular range of Hbs that they doubt they should just let us know so that we…they draw our attention to the fact that maybe we may be having problems with the machine that we do not even know.”*
12	Interview, GH	Clinical staff	*“…they said the Hb was 9.4. but when you look at the face, conjunctiva and everything, I mean, there’s no blood at all. So, we gave 2 units and now she has been discharged this morning.”*
13	FGD, GH	Laboratory staff statement during FGD	*“… lab people will monitor its usage in the sense that maternity, we can give maternity a full pack [pack of POCT]. We want to monitor its usage because they will know how to use it, of course, but if it’s getting finished there they will have to account for it either sense because if we just bring it into the system just like that people will be doing it and some staff can even steal it and take it home; to do it for others for money meanwhile anything can happen so it has to be monitored but it would help.”*
14	FGD, GH	Clinical staff statement during FGD	*“I: How do you feel about the monitoring aspect that the lab is talking about?* *R: The monitoring she is talking about, I agree with her in some part but I disagree with her in a statement she made. We are unique individuals. You don’t know how people would…it is true, some people can take something outside, but I don’t think the ward in charge will just put the thing on the table that everybody can use it. I don’t think so. So, I agree with her but some statement I don’t agree with her.”*
15	FGD, GHC	Clinic staff	*“Aah, after I have done mine and seen that it is negative but still the client is insisting, I’ll let the lab man also come in.”*
16	FGD, GHC	30 y/o, pregnant woman	*“She places it under and sets it to see which colour matches… Please we saw that she was doing it like that, but she didn’t tell us what she was doing …. She did not say anything to us. She just pulled the paper and began to write.”*
17	Interview, GH	Clinical staff	*“No because when the patient comes, they expect to get something. At least if you give them... if nothing at all if you give them paracetamol, if you treat symptomatically without targeting the main cause of disease they would prefer it rather than telling them go, get worse and come back.”*
18	FGD, GH	21 y/o, pregnant woman	*“So, sometimes the fault is from us. Some people when admitted will rush and tell the doctor to discharge them when she is not even well.”*
19	Interview, GH	Clinical staff	*“At times when you do the test and it’s negative, it is nothing, no MPs seen, you can’t give the malaria treatment. If you give, they won’t pay, to be frank. They won’t pay so the protocol here is, when the mother presents the symptoms, then you can start the malaria [treatment].”*
20	FGD, PMC	23 y/o, pregnant woman	*“Please as long as we are human, if you argue with her about taking it, she will be angry. And when you come, what she has to do for you she will be reluctant and the love she has to demonstrate for you, she wouldn’t have that for you.”*
21	FGD, GHC	30y/o, pregnant woman	*“Just as she said. If there was one here and every time you came you knew where your blood level was, when you go home you will take care of yourself in terms of your diet.”*
22	Interview, GHC	Laboratory staff	*“It helps her too. When you are done you will show it to her, telling her that this is the amount of blood I got during the test. Then I will explain the colours for her to see whether her blood level is high or low… It helps them so that if they were not planning on taking the drugs, they take them this time.”*
23	FGD, GHC	Facilitator discussing with pregnant women during FGDs	*“Ok. Now you’ve told me that when you come here you report to the midwife. The midwife takes you through a lot of things and then directs you to the lab. The lab man also pricks your finger and asks you to wait outside after which he gives you your paper when it is ready, for you to take back to the midwife. You usually don’t know what requests for you to do at the lab. The particular disease, you are not aware of.”*

### Current practice for anaemia and malaria testing

All three facilities used mRDTs for testing: the government hospital (GH) as an adjunct to microscopy, the other two (GHC &PMC) as their only form of malaria testing. At the Private Maternity Clinic (PMC), the midwives and health-care assistants used rapid diagnostic tests at the point of care as standard practice, however, midwives sought clarification from the doctor and/or laboratory staff when results conflicted with their clinical judgement. At GH and PMC, the type of mRDT being used could not be established because staff often procured their own from local pharmacies. During FGD with clinical and laboratory staff (GH), it was reported that testing was only for falciparum malaria, therefore when negative, results did not exclude the possibility of non-falciparum malaria. The clinical and laboratory staff during FGDs felt that there was a higher prevalence of non-falciparum malaria in reality than the national estimated prevalence of 7% (Quote 1–2; Table 2).

Although all three facilities were familiar with HCS, none of the facilities used them routinely. PMC had previously used HCS as part of the initial study. Anaemia diagnosis at each of the facilities was undertaken as follows:

- *Government hospital*: Full blood count machine, spectrophotometry, and haematology analyser.
- *Government health centre*: No laboratory anaemia diagnosis carried out due to resource limitations.
- *Private maternity clinic:* Sahli Method of Haemoglobin Estimation

At all three facilities, irregular supply of RDT and HCS were reported during the study. During the preceding study, supplies were regular due to procurement for research activities of the project at the time [9]. Afterwards, HCS was no longer being supplied (which coincides with the time of this study) because the initial project had ended. RDT however continued to be supplied routinely by the Ghana Health Service (GHS). Irregular supply referred to RDT, and by extension to HCS use, as it had not been adopted for use by health procurement services.

All facilities utilised clinical diagnostic methods for both anaemia and malaria irrespective of laboratory findings. Symptoms used to diagnose malaria included: abdominal pain, fever, headache, chills and a bitter taste in patients’ mouth. Signs used to diagnose anaemia included: pallor of conjunctiva, tongue and palms. In general, the clinical judgement determined whether a patient was treated or not, irrespective of the laboratory test result (Quote 3–4; Table 2).

Across all three facilities, training had been sporadic and improvised for the laboratory staff. None of the facilities visited reported having regular and consistent training on anaemia or malaria and their diagnostics. When it did happen, it was reported that one staff member attended training workshops and was expected to disseminate the knowledge across the organisation as appropriate. There were no explicit mechanisms in place for this to be done, causing staff to operationalise guidelines for practice differently. There also seemed to be a limited understanding regarding the rationale behind stipulated guidelines, which meant that staff had inadequate information to identify and/or address the poor practice. The lack of knowledge also led to limitations in considerations for differential diagnosis of malaria and anaemia (Quote 5; Table 2).

### Perceptions about tests for malaria and anaemia

The laboratories reported turnaround times between 2 and 45 min for malaria and 5 and 15 min for anaemia (where done) which they felt was prompt, and up to an hour at peak periods. However, ANC staff at two of the facilities (PMC and GH), felt that results usually took too long to return. The women likewise felt that the testing and consultation process was time consuming.

Regarding accuracy of results, laboratory staff recognised some limitations with the POCT they used but generally felt that the tests were accurate. At GHC in particular where only mRDTs were available to test for malaria, the laboratory staff felt the limitations of mRDTs made them significantly inferior to microscopy for malaria diagnosis. ANC staff expressed concerns that human error also led to inaccuracies in laboratory tests (e.g. mixing up of patient results), despite also having reservations regarding the accuracy of POCT.

### Perceptions of advantages and disadvantages of point of care testing for malaria and anaemia

**Fig. 1:**
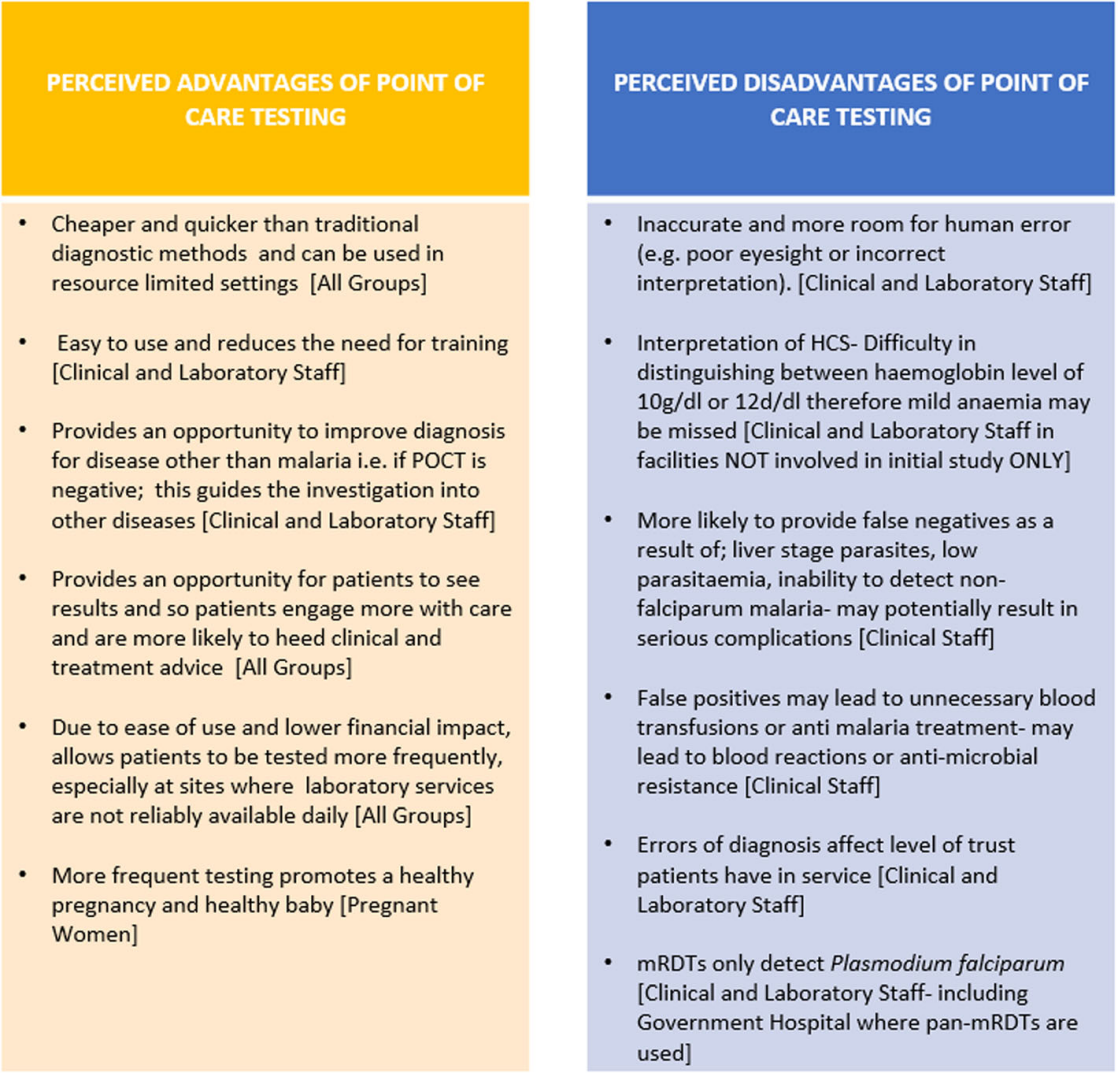
Perceived advantages and disadvantages of Point of Care Testing for malaria and anaemia

A range of advantages and disadvantages about malaria and anaemia POCT were identified by the groups (Fig. 1). The pregnant women did not identify any disadvantages.

Despite identifying a number of disadvantages for the use of POCT, clinical staff were all keen for both the roll out of HCS and change in protocol to recommend that anaemia and malaria POCT be undertaken by all clinical staff, if they were not already doing so. They expressed no concerns regarding the impact of taking on the task on their existing workload. The laboratory staff on the other hand felt that clinical staff would not have the necessary training to safely carry out the task such as use of personal protective equipment that could impact on both staff and patient safety. There were also concerns of accuracy of the result due to human errors that could lead to discrepancies in patient diagnosis (Quote 6; Table 2).

Although the laboratory staff acknowledge the usefulness of POCT especially in emergency cases when laboratory services could not be accessed, they felt that increasing access to POCT for pregnant women could also negatively impact on demand for laboratory services in future. This, they felt, would significantly reduce their workload, and consequently their job security.

### Perceptions and drivers of symptom-based diagnosis

At two out of the three facilities, clinical staff felt that symptom-based diagnosis was quicker and cheaper than other forms of diagnosis because access to their own stock of POCT during consultations was unreliable. Clinical diagnoses were used as a primary method of diagnosis of malaria and anaemia, and to justify treatment in those who had had negative test results from either POCT or other forms of laboratory testing. Staff also perceived that their seniors used symptom-based diagnosis as a default method. Although staff reported to having received guidance from the government and other external agencies that treatment should not be decided from symptom-based diagnosis, in practice this guidance was largely ignored (Quote7; Table 2).

**Fig. 2:**
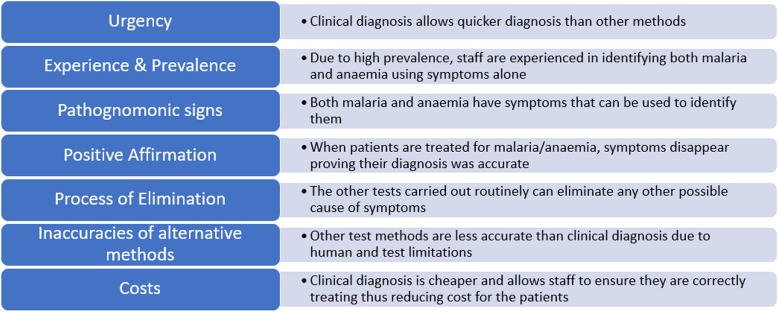
Key drivers and justifications for the use of symptom-based diagnosis by clinical staff

The ANC staff generally felt that symptom-based diagnosis is an effective and efficient means of testing and that each facility had methods in place to ensure appropriate use of it. At PMC and GHC, the staff reported that it was left to senior members of midwifery staff to decide whether to treat for malaria, even when mRDT provided negative test results. Figure 2 outlines key drivers and justifications for the use of symptom-based diagnosis as expressed by the clinical staff.

Laboratory staff highlighted a number of problems associated with symptom-based diagnosis. Primarily, they felt that the symptoms used for diagnosis of anaemia and malaria are not pathognomonic, leading to an over-diagnosis and over-treatment of malaria and, to a lesser extent, anaemia. They felt that this practice increased the risk of anti-microbial resistance to malaria drugs and an overuse of already stretched blood stocks for transfusions (Quote 8; Table 2).

Although laboratory staff attended training about overdiagnosis and overtreatment of malaria and anaemia, there was no formal way of ensuring these messages were disseminated across the facility. Nevertheless, they felt that the clinical staff should already be aware of this and expressed their frustration about being helpless to change behaviour as symptom-based diagnosis was routinely adopted as the diagnostic of choice across the country (Quote 9–10; Table 2).

### Inter-relationships between ANC and laboratory staff, and patients

#### Relationships between ANC and laboratory staff

Observations at all three facilities highlighted a potential lack of effective communication leading to mistrust between laboratory staff and clinical staff. Communication of results between the staff groups was mainly via written result slips, couriered by the patient. GH laboratory staff stated that they regularly requested feedback regarding unusual results in order to check quality of the result, as well as to promptly identify problems with laboratory testing (Quote 11; Table 2).

Notwithstanding, ANC staff felt they could not rely on laboratory diagnostic results alone and therefore often preferred to trust their clinical judgement. They recollected previous evidence to suggest that the laboratory results were often incorrect (Quote 12; table2).

Likewise, laboratory staff did not consider that clinical diagnostic methods to be accurate and felt that clinical staff should not be responsible for managing POCT and their use. The lack of trust and power relations was further displayed during joint focus groups. During FGDs, controversial statements were made by laboratory staff regarding POCT use by clinical staff (Quote 13; Table 2). The clinical staff choose not to voice opinions or challenge speculative comments made by laboratory staff, until they were encouraged by the focus group facilitators (Quote 14; Table 2).

Irrespective of hierarchy and trust concerns impacting staff relationships, clinical and laboratory staff were able to demonstrate cooperation when it came to management of malaria and anaemia in pregnancy (Quote 15; Table 3).

**Table 3:** Key recommendations for effective implementation of point-of-care testing for the diagnosis of malaria and anaemia

**Combating POCTs accessibility/availability issues**	In order to enable staff to rely on POCT, it is essential that supply of the tests is consistent as highlighted by previous studies [1]. Context specific supply chain analyses should be carried out to identify and resolve any bottlenecks.
**Education and training**	Comprehensive training should be developed for healthcare providers who deliver POCT, including clarification of the relative advantages of POCT in comparison to symptom-based diagnosis. We identified needs for training in effective use of the tests to ensure optimal accuracy, strengths and limitations of POCT, as well as problems associated with symptom-based diagnosis of malaria and anaemia.
**Encouraging patient involvement**	Interventions to increase patient awareness of POCTs and their benefits could potentially improve patient involvement. Our findings showed staff felt it may allow the women to become more involved and compliant with healthcare recommendations.
**Supporting the development of effective working relationships between key specialities**	This study highlighted barriers to effective communication between laboratory and clinical staff, and an implicit hierarchical power structure. The use of multidisciplinary group effort, with strong leadership, may be a method in which to combat these barriers [18]. This, plus joint training sessions could ensure key messages can be passed between staff groups, through open and honest communication.
**Development of policy**	Fundamental to these recommendations is the need for effective policy and practical guidance on the diagnosis and treatment of malaria and anaemia in these regions. Although local government recommendations mirrored the WHO guidelines, this was not translated into practice at the health facilities. Staff involvement in the development of local policy is essential to ensure standardisation and understanding of practice.

#### Relationships between ANC/laboratory staff and pregnant women

The pregnant women demonstrated a good understanding of tests that were required during their pregnancy. At the point of laboratory and POCT testing, the women could not identify which tests were being undertaken but this soon became clearer upon receipt of the result from the clinical staff. There was no indication from the women that they ever challenged the lack of information or asked questions (Quote 16; table2).

In contrast, laboratory and clinical staff felt that they did explain the tests that were being carried out to the women prior to testing. They discussed the role that pregnant women played in their diagnosis and treatment which showed variations in the perceived level of control and influence that the women had regarding treatment choices. Although they recognised that pregnant women’s treatment preferences may influence their clinical management, there was a general consensus rejecting the idea that the women should query their treatment. Nonetheless, they felt that the women retained the power to choose whether or not to adhere to recommended treatment, buttressed by the women, who also reported they recognised the role they had to play in their diagnosis and treatment (Quote 17–18; Table 2).

A clinical staff member felt having a negative malaria test as a barrier for treatment adherence. She felt the women would not adhere to treatment if they were aware their test result was negative, which would also lead to reluctance to pay for the test and treatment. As a result, it was reported that the culture was not to test for malaria but to adhere to symptom-based diagnosis and presumptive management (Quote 19; Table 2).

During discussion at FGDs, the women felt that clinical and laboratory staff were the main decision-makers in their treatment and had mixed opinions about the appropriateness of challenging clinical or laboratory staff if they were treating them despite a negative test result, speaking fondly of the clinical staff. However, this often translated into a fear of challenging the clinical staff which they felt could damage their relationship and potentially lead to annoyance of clinical staff (Quote 20; Table 2).

All three stakeholder groups felt that POCT provided an opportunity for the women to see their results, which potentially increased patient engagement thereby improving treatment adherence. The women likewise felt that implementation of POCT by clinical staff would increase their involvement in the decision-making process of their treatment. The women also felt they would be well informed to challenge decisions made by the clinical staff (Quote 21–22; Table 2).

The pregnant women had less interaction with the laboratory staff which was reflected in their perceptions. Although the laboratory staff agreed with clinical staff concerning pregnant women’s perception of POCT use, the laboratory staff who had contacted the pregnant women were male while the clinical staff described in the FGD were mainly female. This could have affected the interactions between the pregnant women and clinical and/or laboratory staff (Quote 23; Table 2.

## Discussion

Point-of-care testing (POCT) is an inexpensive and easy to use form of diagnostic testing and is available for many conditions including anaemia and malaria. A wealth of evidence demonstrates the efficacy of these tests and the superiority of them when compared to symptom-based diagnosis [4–6]. Although ante-natal clinical staff had access to and were aware of POCT and recommendations for its use, this study shows a preference for symptom-based diagnosis by clinical staff, corresponding with previous research [6]. There were also concerns about unreliable supply, as well as the accuracy of POCT in detecting malaria and anaemia. Moreover, the study showed a lack of adequate training and engagement amongst the stakeholders about POCT implementation. The irregular supply of POCT, on top of the quick convenience of symptom-based diagnosis by clinical staff, contributed to POCT underutilisation for both anaemia and malaria. This outlook was not shared by laboratory staff who understandably preferred microscopy for diagnosis, negatively affecting working relationships between the two groups.

In this section we discuss the factors affecting POCT implementation which include unreliability of supply, perceptions of accuracy, perceived benefits of symptom-based diagnosis, and how these affect staff engagement and stakeholder relationships.

### Key factors affecting POCT implementation

#### Unreliable supply of POCT for anaemia and malaria

An important finding in this study was the issue of consistency in the supply of both mRDTs and HCS to the healthcare facilities we visited. The facilities faced regular stock-outs of mRDTs, while HCS was unavailable in all three facilities. Although HCS had been piloted during a previous cluster randomised trial in two out of the three facilities, they were available and consistently supplied only during the trial period. There were also concerns about procurement and regular supply of HCS kits (after the trial period), including the special paper required to use them. Even for malaria, health workers reported often having to resort to purchasing mRDTs privately.

The World Health Organisation’s operational manual for universal access to mRDTs (2011) highlights the importance of rigorous situational analysis prior to the roll-out of the tests to ensure effective uptake [10]. Although this study identifies some of the barriers preventing effective uptake of POCT, a logistical analysis of the supply of POCT could potentially detect bottlenecks in the supply chain. This is fundamental to promoting the use of POCT for malaria and anaemia [11].

#### Perceptions of POCT accuracy

The findings of this study showed concerns regarding the accuracy of POCT by both laboratory and clinical staff. Although there is evidence to suggest limitations with mRDTs in particular due to their inability to detect low parasitaemia, liver stage parasites or non-falciparum malaria [12, 13], the three facilities studied expressed preference for pan-malaria mRDTs which detects non-falciparum malaria. Research also shows that HCS, when used appropriately, is more effective in detecting anaemia when compared to symptom-based diagnosis [6, 14].

The lack of trust in the each other’s competencies to carry out the tests demonstrated by the clinical and laboratory staff could be attributed to a lack of understanding and knowledge, in addition to inadequate training in the use of POCT. This highlights the importance of training of staff emphasising their complementary skills, when promoting POCT. Moreover, research shows that effective training on the use of both HCS and mRDTs can improve the sensitivity and specificity of the test [13]. Clinical and laboratory staff also need to be aware of the consequences of unnecessary treatment of malaria and anaemia such as blood borne viruses from blood transfusions, and anti-malarial resistance due to over-prescription.

#### Perceived benefits associated with symptom-based diagnosis

One of the identified barriers to the uptake of POCTs by clinical staff was their previous perception regarding benefits of symptom-based diagnosis. However, studies demonstrate the cost-effectiveness of proper use of mRDTs, when compared to symptom-based diagnosis across many African populations [14]. One of the problems with symptom-based diagnosis of anaemia is that it does not differentiate well between mild, moderate and severe anaemia, which has implications for their differential management. The clinical staff seemed to rely on signs and symptoms of malaria and anaemia, such as a bitter taste in the mouth and pallor, respectively. These features are nonspecific and could lead to inappropriate treatment of the real cause of illness.

Clinical staff also felt their extensive experience in an anaemia and malaria endemic country equipped them to diagnose and treat malaria and anaemia, usually confirmed by prompt recovery of patients following treatment. This, they felt, was confirmation of their diagnosis. Previous studies suggest that the presumptive prescription of anti-malarials may have deterred staff from exploring differential diagnosis of the symptoms, leading to delayed treatment and higher case fatality rates seen in non-malaria fever [15]. Furthermore, in two out of the three health facilities visited, the health care staff had to procure mRDTs for their patients themselves. The out of pocket expenses as well as the inconsistency of mRDT sources could be a disincentive to the use of POCTs in practice, which may have influenced their responses that seemed to favour the use of symptom-based diagnosis of malaria. This may also be related to the decision of clinical staff to treat for malaria, even when results were negative, in order to recoup the cost of procuring the test kits.

Laboratory staff on the other hand recognised a number of the limitations of symptom-based diagnosis, as well as the inaccuracy when compared with laboratory methods. They were also aware that clinical staff often doubted the diagnostic tests that the laboratory staff undertook and preferentially trusted their own clinical judgement.

#### Staff engagement, training, and implementation of POCT for malaria and anaemia testing

Successful utilisation of POCT requires a change in diagnostic methods and behaviours; this can be achieved with consistent support, training and reinforcement of the new techniques. We did not observe any evidence to suggest that clinical staff had been engaged in the decision to introduce POCT. Junior staff being aware that their supervisors used symptom-based diagnosis as a default method also negatively influenced their adoption of POCT as a reliable diagnostic tool. Studies show the importance of peer influence and leadership when adopting new innovations [16]. Utilising respected clinical staff as peer influencers could lead to an increase in uptake of POCT as standard practice.

Staff across the three facilities were aware that the national government required that patients were tested for malaria prior to treatment, however staff in this study appeared to have insufficient training to carry out the task. Although use of POCT posters had been circulated to laboratories, staff seemed unaware of international guidelines such as those in the WHO’s Global Malaria Programme. Adoption of new clinical practice is always challenging and communication style is important in promoting the uptake of a new practice [16]. Evidence suggests that face-to-face communication is more effective than other forms, and when delivered by someone in a similar profession, it is more likely to have a significant impact [17, 18].

#### Stakeholder relationships

It is essential that all groups using these tests can understand the relative advantage of them compared to symptom-based diagnosis. However, lack of effective communication between the clinical and laboratory staff was observed as a major reason for ineffective implementation of POCT. Clinical staff showed reticence in challenging their laboratory colleagues and struggled to express opinions in group discussions unless directly prompted. This issue was not observed during the interview process and may reflect implicit difference in power relations. The diffusion of good practice amongst these communities relies on the strength of the training and relationships between peers. Given the awareness of laboratory staff about the advantages of POCT, and the limitations of presumptive treatment, it was evident that key messages were not being properly communicated between these two groups. Multi-disciplinary team meetings have been shown to support the development of relationships between different specialities and help open up communication channels in a safe environment [19]. Using the multi-disciplinary approach with effective leadership can ensure that all parties involved have the opportunity to discuss concerns regarding implementation of POCT. This can be achieved through regular and interactive health management team visits to observe staff and provide constructive mentorship and coaching, thereby promoting adherence to guidelines and positive health care practice.

In this study, challenges in stakeholder relationships were extended to the end-users - the pregnant women. There was a largely paternalistic model of clinician-patient relationship, with the women complying with clinicians’ recommendations for fear of annoying or angering them. The interactions between staff and the pregnant women could also have been impacted by the gender roles as the laboratory staff were predominantly men while clinical staff were mainly women. While the pregnant women may find it easier to identify and interact with female clinical staff, the level of interaction could be limited between the pregnant women and the laboratory staff (with whom they also had limited contact) affecting the level of communication between the stakeholder groups.

The awareness of the POCT and their testing process was also lacking among the pregnant women. This inhibits the service users from contributing to the decision-making process regarding their health. However, clinical and laboratory staff did feel that POCT may allow the women to become more involved in their healthcare and therefore more likely to comply with healthcare recommendations. Studies reveal complex relationships between active patient participation, patient adherence, intervention implementation and expected health outcomes in malaria and anaemia management [9, 20, 21]. Patient-centred approaches can have an impact on a healthcare practitioners’ approach to care and this could be considered in the development of any training package for the implementation of POCT.

### Limitations of study

This study was undertaken in a single region of Ghana within three antenatal healthcare facilities, and thus represents a very small proportion of the country and stakeholders utilising POCT for malaria and anaemia diagnosis. However, the study did reach a point of saturation, and while generating new knowledge, it corroborates previous findings. Therefore, we are confident that the key themes identified are relevant to address barriers and enablers for effective implementation of POCT. Another limitation is that the study did not focus on measuring the level of stock outs at that time; therefore, we are unable to give figures on stock outs levels.

### Next steps

There is a wealth of evidence to suggest that there are no ‘magic bullets’ when it comes to effective behaviour change. The Health Foundation state that “developing effective behaviour change interventions likely benefits from theory-based behavioural analysis, an appreciation of context and structured selection of possible interventions with a particular consideration of acceptability and equity” [20]. This study identifies barriers and possible enablers to effective behaviour change in this setting for the purpose of successful implementation of POCT. However, as recommended by WHO [10], it is essential that tailored approaches to implementation within the local context be developed to tackle bottlenecks in the provision of POCT. The key recommendations from this study are presented in Table 3.

## Conclusion

Previous studies have suggested that by involving patients in the use of POCT may empower them to reject unnecessary treatment, where they are well enough to do so [9]. Further studies are required to fully elucidate the role of patients in this setting for the successful implementation of POCT.

Development of standard operating procedures (SOPs) is required to ensure standardised methods of testing and diagnosis. Health services should be supported to ensure that they have clear, evidence-based SOPs and processes in place to ensure that guidelines are followed. Clear efforts to engage both clinical and laboratory staff with the development of, and adherence to SOPs should tie into joint training programmes developed for successful roll out of POCT.

## Supplementary information

**Additional file 1 **Pathways to treatment of anaemia and malaria in antenatal care: focus group discussion guide/participatory method (flowchart) – pregnant women.

**Additional file 2 **Pathways to treatment of anaemia and malaria in antenatal care: in-depth interview topic guide for ANC staff.

**Additional file 3 **Pathways to treatment of anaemia and malaria in antenatal care: in-depth interview topic guide for laboratory managers.

**Additional file 4 **Pathways to treatment of anaemia and malaria in antenatal care: in-depth interview topic guide for laboratory managers.

## Data Availability

The datasets used and analysed during the current study are available from the corresponding author on reasonable request. Interview guide used in data collection was developed for this study and a copy in English language version has been uploaded as a supplementary material [Additional files 1, 2, 3 and 4].

## Abbreviations

ANC: Antenatal care
GH: Government hospital
GHC: Government health centre
GHS: Ghana health service
Hb: Haemoglobin concentration
HCS: Haemoglobin colour scale
mRDT/ RDT: Malaria rapid diagnostic test / rapid diagnostic tests
PMC: Private maternity clinic
POCT: Point of care tests
