# Supplementary material for: Improving the effectiveness of point of care tests for malaria and anaemia: a qualitative study across three Ghanaian antenatal clinics
Source: BMC Health Serv Res. 2020 May 19;20:444. doi: 10.1186/s12913-020-05274-7 (PMC7238731; doi:10.1186/s12913-020-05274-7)
Supplement: Supplementary file 1 — Additional file 1 Pathways to treatment of anaemia and malaria in antenatal care: focus group discussion guide/participatory method (flowchart) – pregnant women. [file 12913_2020_5274_MOESM1_ESM.docx]

**Pathways to treatment of anaemia and malaria in antenatal care:
Focus group discussion guide/participatory method (flowchart) – PREGNANT WOMEN**

- Welcome and thank you for participating
  - Introductions
- Purpose and explanation of the session:

The purpose of this session is to explore how malaria and anaemia are diagnosed currently from the perspective of you as pregnant women. We would also like your views on point-of-care testing for the diagnosis of malaria and anaemia in an antenatal context. Point-of-care tests are tests that are performed by non-laboratory healthcare professionals close to the patient. We will explain this in more detail later in the session. We will use the information you give to help produce a list of recommendations for the scale-up of point-of-care testing such as malaria rapid diagnostic tests and the haemoglobin colour scale.

For this project we will be using a combination of group sessions like this one and one-to-one interviews to hopefully allow us to explore the topics in general whilst also gaining more in-depth information from individual experiences.

Group sessions and interviews are also are also being undertaken with antenatal clinic staff and laboratory staff (responsible for the analysis of blood samples taken from pregnant women for things such as malaria and anaemia). We will collate information from all of these sessions and bring this information for further discussion at a final group discussion with another selection of pregnant women. All information will be anonymised where appropriate.

- Highlight issues of confidentiality and the fact that the research is entirely voluntary
- Ask permission to record
- Obtain consent
- Logistics
  - Session will last about one hour
  - Feel free to move around
  - Where is the bathroom? Exit?
  - Help yourself to refreshments
- Ground Rules:
  - Everyone should try to participate.
  - Information provided in the focus group must be kept confidential
  - Stay with the group and please don’t have side conversations
  - Turn off mobile phones if possible
- Any questions?
- Introductions of participants - go around the room
- Discussion Questions:

1. Flowchart: process for diagnosis of malaria and anaemia in pregnant women. Work together to show what happens during your visit to the ANC clinic in terms of malaria and anaemia testing.
2. Let’s discuss the current process for the diagnosis of malaria and anaemia within the ANC. What works well within the current system?
3. Can you identify any areas where the system doesn’t work so well?

*At this stage introduce the idea of POCT (specifically malaria Rapid Diagnostic Tests and Haemoglobin colour scale). Are the group familiar with them? If not describe the concept.*

1. If more antenatal clinics introduced mRDTs/HCS, what may be the effects of this on the ANC/laboratory/patient?
   - Prompts for discussion
     - Awareness of a need for change
     - Perceptions of clinical diagnosis
     - Perceptions of laboratory testing – accurate?
     - Perceptions of RDT accuracy
     - Logistics in the use of RDTs
     - Problems for the women if RDTs were used more frequently –trust?
     - Perceptions of patient preferences re: diagnosis of malaria and anaemia
     - Influence of peers?
