# Supplementary material for: Improving the effectiveness of point of care tests for malaria and anaemia: a qualitative study across three Ghanaian antenatal clinics
Source: BMC Health Serv Res. 2020 May 19;20:444. doi: 10.1186/s12913-020-05274-7 (PMC7238731; doi:10.1186/s12913-020-05274-7)
Supplement: Supplementary file 2 — Additional file 2 Pathways to treatment of anaemia and malaria in antenatal care: in-depth interview topic guide for ANC staff. [file 12913_2020_5274_MOESM2_ESM.docx]

**Pathways to treatment of anaemia and malaria in antenatal care:
In-depth interview topic guide for ANC staff**

**Introductions:**

- Explain who we are
- Explain what the research is about (use information sheet so as to keep it simple)
  - This is not a test and I just want to know your thoughts and opinions, nothing you say will be considered right or wrong.
  - I want to explore people’s views on your views on point-of-care testing for the diagnosis of malaria and anaemia in an antenatal context. Point-of-care tests are tests that are performed by non-laboratory healthcare professionals close to the patient. We would like input from individuals throughout the antenatal clinic and will use this information to help produce a list of recommendations for the scale-up of point-of-care testing such as malaria rapid diagnostic tests and the haemoglobin colour scale ( a point-of-care test for anaemia).
- Highlight issues of **confidentiality** and the fact that the research is entirely voluntary
- Ask permission to record
- Obtain consent

**Ice-breaker questions:**

- Please can you confirm your job role
- How long have you worked at this clinic for?

**Malaria Diagnosis**

- Please tell me about how malaria is currently diagnosed in your clinic?

**Prompts**

- - Laboratory testing or POCT?
  - Clinical diagnosis?
  - How do you choose which method if there are multiple options available?
- What works well with this system?

**Prompts**

- - Accuracy of results? Is this a perception of accuracy or is there evidence?
  - Time taken for results? Max/minimum/usual?
- What could be improved?

**Prompts**

- - Accuracy of results? Is this a perception of accuracy or is there evidence?
  - Time taken for results? Max/minimum/usual?
- What is the involvement of laboratories in this systems?
- How are test results communicated with you by laboratories?

**Prompts**

- Urgent results
- How do you request tests from laboratories?

**Prompts**

- Form of communication – telephone, written?
- Time taken for response
- Please tell me about the pathway to treatment of malaria in the clinic

**Prompts**

- - Treatment on clinical diagnosis alone? i.e prescribing without diagnosis – Perceptions on this.
  - If treat without diagnosis, why?
    - Influence of peers? Influence of patients?

**Anaemia Diagnosis**

- Please tell me about how anaemia is currently diagnosed in your clinic currently?

**Prompts**

- - Laboratory testing or POCT?
  - Clinical diagnosis?
  - How do you choose which method if there are multiple options available?
- What works well with this system?

**Prompts**

- - Accuracy of results? Is this a perception of accuracy or is there evidence?
  - Time taken for results? Max/minimum/usual?
- What could be improved?

**Prompts**

- - Accuracy of results? Is this a perception of accuracy or is there evidence?
  - Time taken for results? Max/minimum/usual?
- What is the involvement of laboratories in this systems?
- How do you communicate with laboratories?

**Prompts**

- Urgent results
- Test requests
- Please tell me about the pathway to treatment of anaemia in the clinic

**Prompts**

- - Treatment on clinical diagnosis alone? i.e prescribing without diagnosis – Perceptions on this.
  - If treat without diagnosis, why?
    - Influence of peers? Influence of patients?

*At this stage introduce the idea of POCT (specifically malaria Rapid Diagnostic Tests and Haemoglobin colour scale). Is the interviewee familiar with them? If not describe the concept.*

- Do you currently use POCT?

If already use POCT (repeat for anaemia and malaria):

- What POCT do you currently use?
- How do these work in conjunction with other testing methods?

**Prompts**

- - Clinical testing and laboratory testing
  - If POCT does not agree with clinical diagnosis what is the protocol
  - Do test results get followed up by further laboratory tests? In all cases or just some?
- How do you get supplies of POCTs?
  - Payment in comparison to other testing?
  - Are there regular stock outs and if so how many and how long do they last?
- How well does POCT work for diagnosis of malaria/anaemia?

**Prompts**

- - Perception of POCT accuracy
  - Any evidence for their perceptions
  - Patient perceptions of POCT
- How do you benefit from using POCT?

**Prompts**

- - Speed?
  - Comparison of this with clinical diagnosis
- What are the disadvantages of using POCT?

**Prompts**

- - Accuracy of POCT
  - Logistics of POCT

If don’t already use POCT:

- What do you know about POCT?

**Prompts**

- - Accuracy of POCT
  - Logistics of POCT
  - Personal perceptions of POCT
  - Have they heard of POCT working in other clinics?
  - Ideas of perceptions of patients
- How do you feel POCT may work in your ANC setting?

**Prompts**

- - Benefits
  - Disadvantages
- What would you perceive would be the steps required to effectively roll-out POCT?
- What may be the barriers to the roll-out of POCT?
- How could/should quality of POCTs be checked?
- Any other questions?

Thank informant for participating. Explain the next step i.e. FGD for solutions. Ask for involvement. Explain how results will be disseminated back to staff.
