# Supplementary material for: Improving the effectiveness of point of care tests for malaria and anaemia: a qualitative study across three Ghanaian antenatal clinics
Source: BMC Health Serv Res. 2020 May 19;20:444. doi: 10.1186/s12913-020-05274-7 (PMC7238731; doi:10.1186/s12913-020-05274-7)
Supplement: Supplementary file 3 — Additional file 3 Pathways to treatment of anaemia and malaria in antenatal care: in-depth interview topic guide for laboratory managers. [file 12913_2020_5274_MOESM3_ESM.docx]

**Pathways to treatment of anaemia and malaria in antenatal care:
In-depth interview topic guide for Laboratory managers**

**Introductions:**

- Explain who we are
- Explain what the research is about (use information sheet so as to keep it simple)
  - This is not a test and I just want to know your thoughts and opinions, nothing you say will be considered right or wrong.
  - I want to explore people’s views on your views on point-of-care testing for the diagnosis of malaria and anaemia in an antenatal context. Point-of-care tests are tests that are performed by non-laboratory healthcare professionals close to the patient. We would like input from individuals throughout the laboratory, including scientists, technicians and managers. We will use this information to help produce a list of recommendations for the scale-up of point-of-care testing such as malaria rapid diagnostic tests and the haemoglobin colour scale ( a point-of-care test for anaemia).
- Highlight issues of **confidentiality** and the fact that the research is entirely voluntary
- Ask permission to record
- Obtain consent

**Ice-breaker questions:**

- Please can you confirm your job role
- How long have you worked at this laboratory for?

**Malaria Diagnosis**

- Please tell me about the process of malaria diagnosis in the laboratory currently?

**Prompts**

- - From sample receipt to result release
  - TAT
- What works well with this system?

**Prompts**

- - Accuracy of results?
  - TAT
- What could be improved?

**Prompts**

- - Accuracy of results?
    - Training of laboratory staff
    - Availability of equipment/reagents
  - Time taken for results?
- What quality assurance and quality control processes do you have in place in the laboratory for anaemia testing? (May need to take contact details to follow up these questions)
- How do you communicate with antenatal clinics?

**Prompts**

- Urgent results
- Test requests
- General test results
- How do antenatal clinics utilise the laboratory services for malaria testing?
  - Would clinical diagnosis be done?
  - Perceptions of accuracy
  - Perceptions of relationships with antenatal clinic staff

**Anaemia Diagnosis**

- Please tell me about the process of anaemia diagnosis in the laboratory currently?

**Prompts**

- - From sample receipt to result release
  - TAT
- What works well with this system?

**Prompts**

- - Accuracy of results?
  - TAT
- What could be improved?

**Prompts**

- - Accuracy of results?
    - Training of laboratory staff
    - Availability of equipment/reagents
  - Time taken for results?
- What quality assurance and quality control processes do you have in place in the laboratory for anaemia testing? (May need to take contact details to follow up these questions)
- How do you communicate with antenatal clinics?

**Prompts**

- Urgent results
- Test requests
- How do antenatal clinics utilise the laboratory services for anaemia testing?
  - Would clinical diagnosis be done?
  - Perceptions of accuracy
  - Perceptions of relationships with antenatal clinic staff

**Introduce POCT – HCS and RDTs**

- What do you know about POCT?

**Prompts**

- - Accuracy of POCT
  - Logistics of POCT
  - Personal perceptions of POCT
  - Have they heard of POCT being utilised in antenatal clinics?
  - Ideas of perceptions of patients
  - Perceptions of POCT accuracy
  - Comparison of costs with comparison to laboratory testing
  - Stockouts
- Does the laboratory have any involvement in POCT?
  - Monitoring?
  - Training?
  - Supply?
- What may be the benefits of using POCT?

**Prompts**

- - Speed?
  - Comparison of this with clinical diagnosis
- What are the disadvantages of using POCT?

**Prompts**

- - Accuracy of POCT
  - Logistics of POCT
- What may be the barriers to the roll-out of POCT?
- How may the up-scale of POCT testing in an ANC setting effect the laboratory?

**Prompts**

- - Funding?
  - Overall utilising of laboratory services
  - Changes to the use of other testing services
- What would you perceive would be the steps required to effectively roll-out POCT?
- Any other questions?

Thank informant for participating. Explain the next step i.e. FGD for solutions. Ask for involvement. Explain how results will be disseminated back to staff.
